# Comparison of influenza viral load in nasopharyngeal and midturbinate swabs

**DOI:** 10.1111/irv.13253

**Published:** 2024-01-21

**Authors:** Corrado Bernasconi, Laurie Katugampola, Steffen Wildum

**Affiliations:** ^1^ F. Hoffmann‐La Roche Ltd. Basel Switzerland; ^2^ Roche Products Ltd. Welwyn Garden City UK; ^3^ Present address: Limites Medical Research Ltd. Vacallo Switzerland

**Keywords:** CENTERSTONE, influenza virus, midturbinate swab, nasopharyngeal swab, specimen type, viral load

## Abstract

Different specimen types are used for influenza diagnosis but comparative data for viral loads from different swab types are limited. We compared influenza viral loads (determined by quantitative reverse transcription polymerase chain reaction [RT‐PCR]) in 93 paired midturbinate and nasopharyngeal swab aliquots from influenza infected patients enrolled in a phase 3 randomized‐controlled study with the objective of maximizing the number of swabs available for sequence analysis. Midturbinate swabs yielded a 53% lower viral load versus nasopharyngeal swabs, and this difference was similar for influenza A and B. These data suggest that nasopharyngeal swabs might be preferred in diagnostic settings when obtaining higher viral load is important.

## 1 INTRODUCTION

Influenza is a major cause of acute respiratory illness and accounts for around 3–5 million cases of severe disease and up to 650,000 deaths globally each year.^1^ The detection of influenza virus in upper respiratory tract samples from ill individuals is the most commonly used method to identify influenza virus infection. A number of respiratory specimen types may be used, including swabs, brush, aspirate, and wash, and specimens may be collected from numerous sites, including the anterior and posterior nasopharynx, oropharynx, and nares. The nasopharyngeal swab (NPS) is traditionally considered the “gold standard” with the highest sensitivity for influenza virus detection; however, this swab type is less comfortable for patients compared with less‐invasive swabs such as the midturbinate swab (MTS), and obtaining these specimens requires specially trained personnel. Comparative data for sensitivity of different swab types used for influenza diagnosis, especially comparing quantified viral loads, are still limited. Here, we report on a subanalysis of data from an ongoing clinical study and compare influenza viral loads in paired NPS and MTS samples.

## 2 MATERIALS AND METHODS

This is a subanalysis of the ongoing double‐blind, multicenter, phase 3 CENTERSTONE study (NCT03969212) evaluating baloxavir marboxil versus placebo for the prevention of influenza transmission within households. The analysis was performed primarily for study planning purposes to inform a potential change in sampling strategy for a maximum viral sequencing yield. Ethics approval for CENTERSTONE was obtained from the institutional review board or independent ethics committee for each participating center. The study includes 5 to 64 year‐old patients who provided informed consent and who presented with suspected influenza (fever or any other symptoms that developed within 48 h of inclusion) but were otherwise healthy.

The analysis used NPS and MTS samples obtained prior to treatment as part of the study procedures. Per study guidance, an MTS sample was taken using an adult midturbinate nylon flocked swab (Copan FLOQSwabs® 56380CS01) from one nostril immediately followed by an NPS sample taken from the other nostril using a minitip nylon flocked swab (Copan FLOQSwabs® 501CS01). Collected swabs were placed into tubes filled with universal transport medium (Copan UTM® 330C) and stored in refrigerated conditions (2–8°C). An aliquot of the NPS sample was utilized immediately for local influenza testing by polymerase chain reaction (PCR) or rapid influenza diagnostic test. Within 12 h from sample collection, the remaining NPS sample and the MTS sample were shipped refrigerated to the central laboratory processing center who received the samples within 36 h from sample collection. Samples were then aliquoted and stored at −80°C until central laboratory testing. In case of positivity for influenza A or B in the local test performed on the NPS, the viral load was determined in both samples by the central laboratory (Viroclinics‐DDL, Rotterdam, The Netherlands) using the MagNA Pure 96 system (Roche) for viral RNA purification and a proprietary influenza matrix gene quantitative RT‐PCR assay (lower limit of quantitation for influenza A: 2.79 log_10_ virus particles [vp]/mL; influenza B: 2.63 log_10_ vp/mL) with the purpose of determining differences between the two procedures, including the proportion of samples with viral loads above 10,000 vp/mL, which was the typical cut‐off for samples to be submitted to sequencing at our central laboratory. This reflects a high probability of successful Sanger sequencing for samples exceeding that viral load.

The statistical analysis was exploratory and primarily based on descriptive methods. Statistical tests were conducted at the 5% significance level without correction for multiplicity. We used the Wilcoxon rank‐sum and signed‐rank test for unpaired and paired samples of continuous values, respectively, and McNemar's test for paired binary data. The sample size was not determined prospectively but reflected the number of study subjects that, at the time of the analysis, had both samples assessed with the most recent version of the central quantitative RT‐PCR assay.

## 3 RESULTS

The analysis was based on 104 patients (mean age 28 years, range 5–63 years, 55% females) who provided both types of samples. All patients presented with at least one of the symptoms of influenza, which had started within the last 48 h and in 30% of cases within the last 24 h. Most samples (81%) were collected during the 2022/2023 influenza season, and 16% and 3% of the samples originated from the 2019/2020 and 2021/2022 season, respectively. Influenza A and B viruses were detected in 59% and 41% of patients, respectively. One case in which influenza A virus was detected only in the NPS, and influenza B virus in both specimens, was classified as B in the analysis.

**FIGURE 1:**
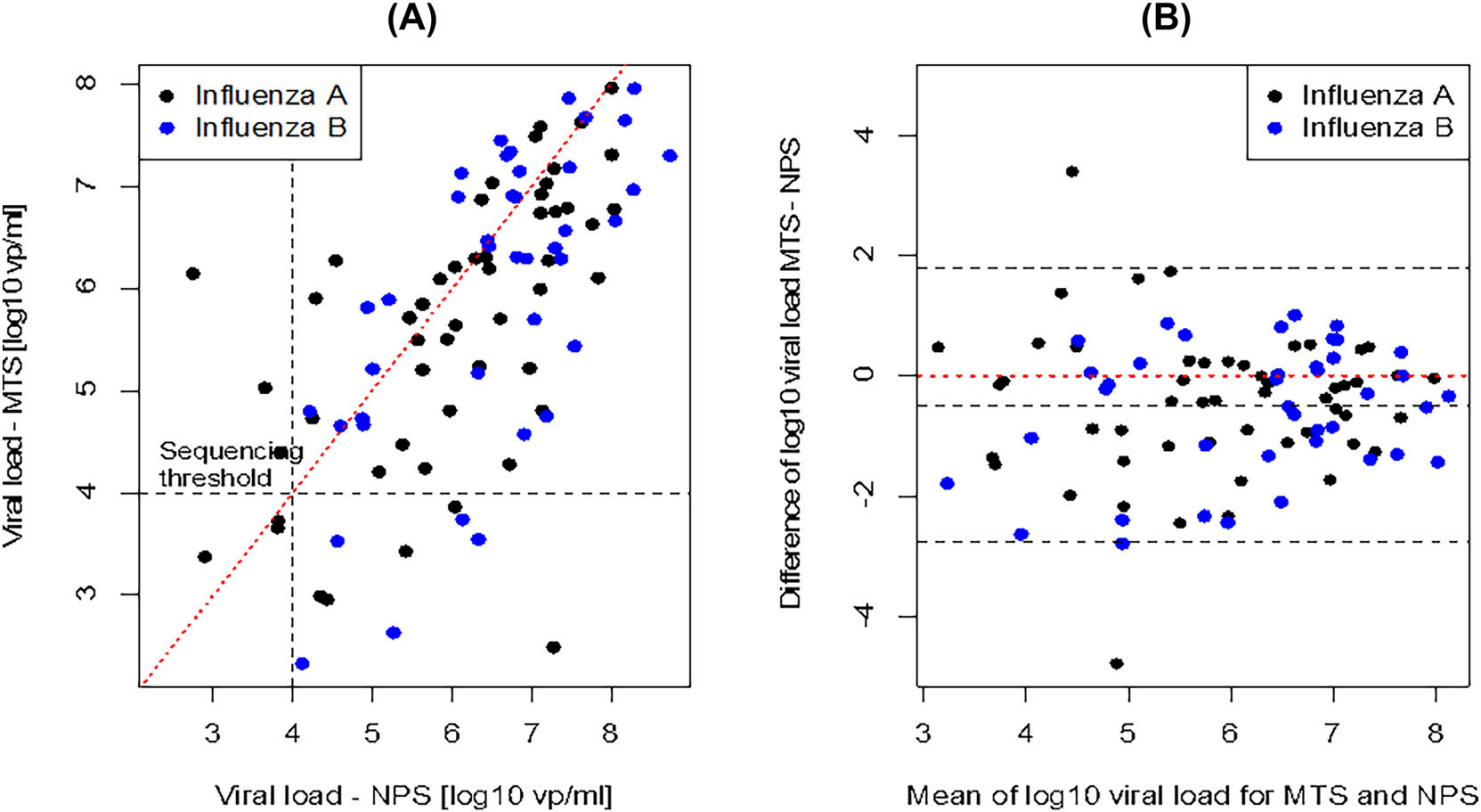
(A) Scatterplot of viral loads for midturbinate swab (MTS) versus nasopharyngeal swab (NPS) on a log_10_ scale. The horizontal and vertical dotted lines correspond to 10,000 virus particles (vp)/mL (typical threshold for sequencing at the central laboratory). The dotted red line is the main diagonal. (B) Bland–Altman plot of viral loads in log_10_ vp/mL for MTS versus NPS. The three black dotted lines represent the mean difference and the 95% confidence limits of agreement.

NPS and MTS returned a positive central result in 95% and 92% of cases, respectively. Median viral loads were 2.3 and 1.1 million vp/mL (6.37 and 6.04 vp/mL on a log_10_ scale) for NPS and MTS, respectively (Table 1). In a paired analysis (Table 2 and Figure 1), NPS provided significantly higher viral load versus MTS (*p* = 0.0002). The relative viral load difference based on median values corresponded to 53% lower viral load in MTS versus NPS. The difference between NPS and MTS was of similar magnitude and remained significant in separate analyses by influenza type: For patients with influenza A, the difference in log_10_ units was 0.27 vp/mL (*p* = 0.0083), and for influenza B, the difference was 0.31 vp/mL (*p* = 0.0079). For influenza A virus samples with available subtype and viral load information (*n* = 49), the two subtypes H1N1 and H3N2 behaved similarly; the same was the case for samples from adults (age ≥18 years) and from children/adolescents (age 5–17 years).

**TABLE 1:** Viral load in nasopharyngeal swab (NPS) and midturbinate swab (MTS).

	*n*	Min value	1st quartile	Median	Mean	3rd quartile	Max value	*SD*	Missing value (not quantifiable)
NPS	99	2.33	5.24	6.37	6.11	7.16	8.74	1.42	5
MTS	96	2.33	4.73	6.04	5.72	6.88	7.96	1.42	8

*Note*: Viral load values are shown in log_10_ virus particles per milliliter.

Abbreviation: *n*, number of samples; *SD*, standard deviation.

**TABLE 2:** Viral load difference in midturbinate swab (MTS) versus nasopharyngeal swab (NPS) by influenza type and age (analysis of paired samples).

	*n*	Min value	1st quartile	Median	Mean	3rd quartile	Max value	*SD*	Missingvalue (not quantifiable for at least one swab type)	Wilcoxon signed‐rank test for no difference MTS vs. NPS
Influenza A	53	−4.79	−1.11	−0.27	−0.43	0.24	3.4	1.23	8	*p* = 0.0083
A/H3	32	−2.45	−1.12	−0.29	−0.28	0.31	3.4	1.25	4	*p* = 0.1386
A/H1	17	−2.18	−0.9	−0.27	−0.46	−0.04	0.52	0.73	2	*p* = 0.0267
Influenza B	40	−2.79	−1.31	−0.31	−0.56	0.23	1.01	1.08	3	*p* = 0.0079
Age ≥ 18 years	70	−2.63	−1.15	−0.28	−0.42	0.24	3.4	1.06	7	*p* = 0.0015
Age < 18 years	23	−4.79	−1.26	−0.44	−0.7	0.18	1.61	1.44	4	*p* = 0.0605
All samples	93	−4.79	−1.16	−0.29	−0.49	0.24	3.4	1.16	11	*p* = 0.0002

*Note*: Viral load values are shown in log_10_ virus particles per milliliter.

Abbreviations: *n*, number of evaluable samples; *SD*, standard deviation.

For both procedures, most samples were above the cut‐off for sequencing with slightly higher proportions for NPS versus MTS (86.5% vs. 78.8%, *p* = 0.099).

## 4 DISCUSSION

NPSs are widely used in influenza diagnosis because of their high sensitivity for viral detection, but they are uncomfortable for patients. In the CENTERSTONE study, NPS but also the less‐invasive and easier‐to‐obtain MTS were used to reduce the burden for the patient when multiple samples were taken for local and central testing. We sought to compare the viral loads obtained from MTS and NPS to determine if both specimens are equally applicable for viral sequencing. Available data in the literature are very limited, with one study reporting higher (1.3 to 2.6 copies/mL on a log_10_ scale) influenza A viral loads in nasopharyngeal aspirates compared with nasal and throat swabs,^2^ and most other studies only comparing qualitative sensitivity for different specimen types.^3^, ^4^, ^5^ One study found similar viral loads in NPS and MTS from infants—but for respiratory syncytial virus,^6^ and results might be different for different viruses. To our knowledge, this is the first study comparing quantitative viral loads in NPS and MTS for influenza A and B.

We found that viral loads were on average higher in NPS versus MTS, both for influenza A and B. Our analysis included samples from symptomatic patients that were positive in a first influenza test (PCR or rapid influenza diagnostic test) performed at different local laboratories on the NPS sample. This limits the ability to compare the accuracy of the overall diagnostic procedure, and in particular its sensitivity, for the two types of swabs. In line with the selection of locally positive samples, centrally, we found a high proportion of positive samples, with slightly higher positivity rates for NPS. In general, it is reasonable to expect for higher viral loads to result in high true positive rates and high sensitivity for the detection of influenza. This can be particularly important in situations with presumably low viral loads, for example, in screening investigations in oligosymptomatic or asymptomatic patients, in patients under antiviral treatment, or in experimental studies following viral shedding over time. Of note, the selection of patients for the CENTERSTONE study and this exploratory investigation targeted recent (≤48 h) symptom development in untreated patients, that is, presumably high viral loads.

The sample size, although not prospectively defined, can be considered adequate in relation to the main study objectives, as it permitted the distinction between viral loads from the two sampling approaches overall as well as within each of the two influenza virus types.

In this study, NPS provided significantly higher viral loads compared with MTS, as well as nonsignificant higher proportions of positive and sequenceable samples. In conclusion, in cases where the influenza viral load detected by the diagnostic procedure is relevant, NPS might be preferable over MTS.

## AUTHOR CONTRIBUTIONS

**Corrado Bernasconi:** Conceptualization; formal analysis; writing—original draft; writing—review and editing. **Laurie Katugampola:** Conceptualization; investigation; writing—original draft; writing—review and editing. **Steffen Wildum:** Conceptualization; investigation; writing—original draft; writing—review and editing.

## CONFLICT OF INTEREST STATEMENT

C. B. has received consulting fees as a contractor for F. Hoffmann‐La Roche Ltd. during the execution of this work. L. K. and S. W. are employees of, and own stocks in, F. Hoffmann‐La Roche Ltd.

## Data Availability

The data that support the findings of this study are available from the corresponding author upon reasonable request. For up to date details on Roche's Global Policy on the Sharing of Clinical Information and how to request access to related clinical study documents, see here: https://go.roche.com/data_sharing. Anonymized records for individual patients across more than one data source external to Roche cannot, and should not, be linked due to a potential increase in risk of patient reidentification.
